# Saquinavir: From HIV to COVID-19 and Cancer Treatment

**DOI:** 10.3390/biom12070944

**Published:** 2022-07-05

**Authors:** Mariana Pereira, Nuno Vale

**Affiliations:** 1 OncoPharma Research Group, Center for Health Technology and Services Research (CINTESIS), Rua Doutor Plácido da Costa, 4200-450 Porto, Portugal; mariana.m.pereira2097@gmail.com; 2 Institute of Biomedical Sciences Abel Salazar (ICBAS), University of Porto, Rua de Jorge Viterbo Ferreira 228, 4050-313 Porto, Portugal; 3 CINTESIS@RISE, Faculty of Medicine, University of Porto, Alameda Professor Hernâni Monteiro, 4200-319 Porto, Portugal; 4 Department of Community Medicine, Information and Health Decision Sciences (MEDCIDS), Faculty of Medicine, University of Porto, Rua Doutor Plácido da Costa, 4200-450 Porto, Portugal

**Keywords:** saquinavir, drug repurposing, HIV, COVID-19, cancer

## Abstract

Saquinavir was the first protease inhibitor developed for HIV therapy, and it changed the standard of treatment for this disease to a combination of drugs that ultimately led to increased survival of this otherwise deadly condition. Inhibiting the HIV protease impedes the virus from maturing and replicating. With this in mind, since the start of the COVID-19 outbreak, the research for already approved drugs (mainly antivirals) to repurpose for treatment of this disease has increased. Among the drugs tested, saquinavir showed promise in silico and in vitro in the inhibition of the SARS-CoV-2 main protease (3CLpro). Another field for saquinavir repurposing has been in anticancer treatment, in which it has shown effects in vitro and in vivo in several types of cancer, from Kaposi carcinoma to neuroblastoma, demonstrating cytotoxicity, apoptosis, inhibition of cell invasion, and improvement of radiosensibility of cancer cells. Despite the lack of follow-up in clinical trials for cancer use, there has been a renewed interest in this drug recently due to COVID-19, which shows similar pharmacological pathways and has developed superior in silico models that can be translated to oncologic research. This could help further testing and future approval of saquinavir repurposing for cancer treatment.

## 1. Introduction

The human immunodeficiency virus (HIV) is a retrovirus with two types, with HIV-1 acting as the main cause of acquired immunodeficiency syndrome (AIDS) globally since it is the most infectious of the two [1]. As of 2020, 37.7 million people had been living with HIV according to UNAIDS [2]. AIDS involves an acute acquired deficiency of the immunity mediated by cells, which leaves the diseased person susceptible to opportunistic infections and neoplasms [3,4]. 

The HIV has two copies of single-stranded molecule RNA. One of the most important genes of the HIV is the *pol* gene, which encodes two enzymes essential for viral infection: (i) a protease that cleaves protein precursors and (ii) a reverse transcriptase (RT) that transforms RNA into DNA that can be incorporated into the host’s DNA [1]. This virus accumulates in lymphoid tissues, creating viral reservoirs, with its main cellular target being memory CD4^+^ T cells that, when infected, become latently resting and even decrease in the total number. Other cells that become dysregulated by the HIV include B cells, CD8^+^ T cells, nonlymphoid cells, and natural killer cells, which, combined with the previously mentioned depletion and dysregulation of CD4^+^ T cells, leads to a deficiency in the immune response to both HIV and other pathogens [5]. 

Nowadays, treatment of the HIV allows patients to maintain immunologic function by decreasing viral replication, which helps in decreasing both the mortality rates, with higher life quality and expectancy, and the transmission rates, with a decrease of over 90% in sexual transmission of the HIV. The standard of care for HIV infection is a combination of three drugs, two nucleoside analog RT inhibitors (NRTIs) and either a protease inhibitor (PI), a non-nucleoside RT inhibitor (NNRTIs), or an integrase inhibitor, taken orally every day. Alteration of this regimen can happen if (i) it was effective and caused virologic suppression, so treatment is switched to a less potent one, with the goal of maintaining viral suppression, or (ii) it was not effective, with consequent virologic failure (detection of high amounts of HIV RNA), calling for a reevaluation of the treatment regimen [6].

## 2. Saquinavir for the HIV

**Figure 1:**
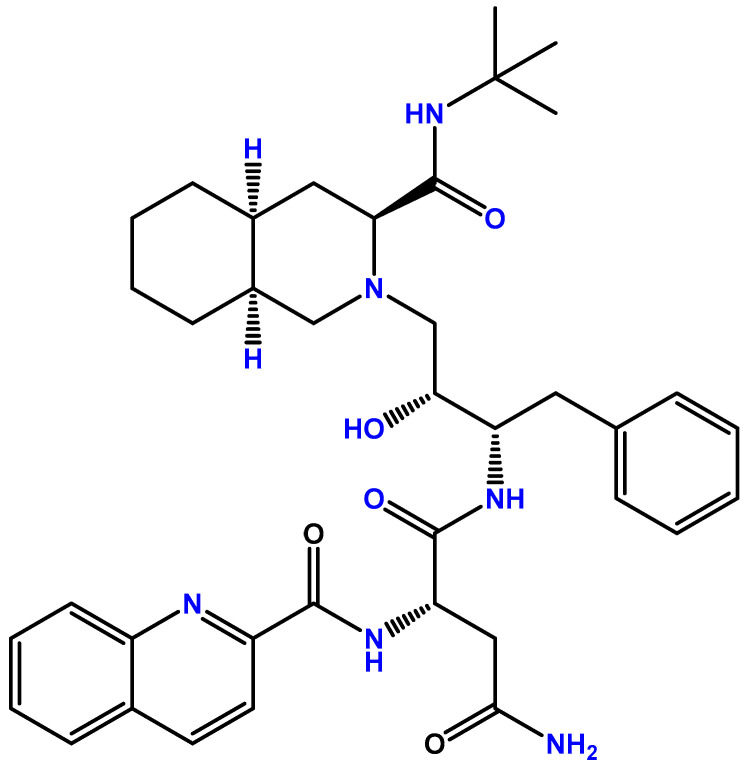
Chemical structure of saquinavir (developed with ChemDraw^®^, a chemical drawing software, https://chemdrawdirect.perkinelmer.cloud/js/sample/index.html, accessed on 5 June 2022).

Saquinavir was the first PI approved for HIV treatment. This drug was first approved by the US Food and Drug Administration (FDA) in 1995 [7] and by the European Medicines Agency (EMA) in 1996 [8] under the name Invirase^®^, produced by Hoffmann-LaRoche. The chemical structure of saquinavir can be found in Figure 1.

This PI was indicated for use in combination with NRTIs already in existence since the combination of drugs was starting to become the standard of treatment for HIV patients, which also caused costs of therapy to increase [7]. In terms of in vitro cytotoxic results, the FDA registered an IC_50_ of 1–30 nM and an IC_90_ of 5–80 nM in acutely and chronically HIV-1-infected peripheral blood lymphocytes as well as monocytic and lymphoblastoid cells. This IC_50_ value had an increase to around 37.7 ± 5 nM when in presence of human serum, a fourfold increase in comparison with the previous values. When combined with several reverse transcriptase inhibitors, such as zidovudine, saquinavir added to a synergistic effect, which was not followed by increased toxicity, an effect also noted in the combination of saquinavir with other PIs, like lopinavir. Direct antiviral activity against HIV-1 and HIV-2 was obtained at IC_50_ ranging from 0.9–2.5 nM and 0.25–14.6 nM, respectively [9].

Bioavailability of saquinavir alone is only around 4% in patients receiving an oral dose of 600 mg in a fed state, a low percentage that is probably associated with a mix of elevated first-pass metabolism and incomplete absorption [6]. Effective treatment in naïve and experienced patients was established with areas under the curve (AUCs) of 20,000 ng·h/mL for both but with minimal concentrations (C_min_) of 50 and 100 ng/mL, respectively. Saquinavir is then distributed into tissues, and 97% of it binds to plasma proteins (with a maximum of 30 μg/mL), with a volume of distribution of 700 L after a 12 mg intravenous dose. Cerebrospinal fluid concentrations are negligible, indicating poor distribution in the brain [10]. In terms of metabolism, it was discovered that saquinavir is highly oxidized in human small intestines into various mono- and dihydroxylated inactive metabolites by the enzyme CYP3A4 of the cytochrome P450 complex. This first-passage metabolism is the main cause for the poor oral bioavailability of this PI when taken alone, as previously mentioned [11]. Another factor that is also responsible for poor oral bioavailability is that saquinavir is a substrate of the multidrug transport system P-glycoprotein (P-gp) which, when present, leads to a decrease in the accumulation of intracellular saquinavir, as well as poor blood–brain barrier penetration [12]. Because of these two factors, saquinavir is used in combination with ritonavir, which is also a PI and has a strong inhibitory effect on the CYP3A4 isoenzyme and the P-gp transporter, increasing effectively the bioavailability of saquinavir and, consequently, its effect on HIV-infected cells without increased toxicity to the patient [13]. Saquinavir is mostly eliminated through feces, with a percentage of 88% when taken orally and 81% when taken intravenously, both after 5 days. In the plasma and after oral ingestion, only 13% of the mass is unchanged saquinavir, with the rest being its metabolites, while when taken intravenously, around 66% of the mass is unchanged saquinavir, a testament to the heavy first-passage metabolism saquinavir endures. This PI also has a rapid systemic clearance and a mean residence time of 7 h [9].

**Figure 2:**
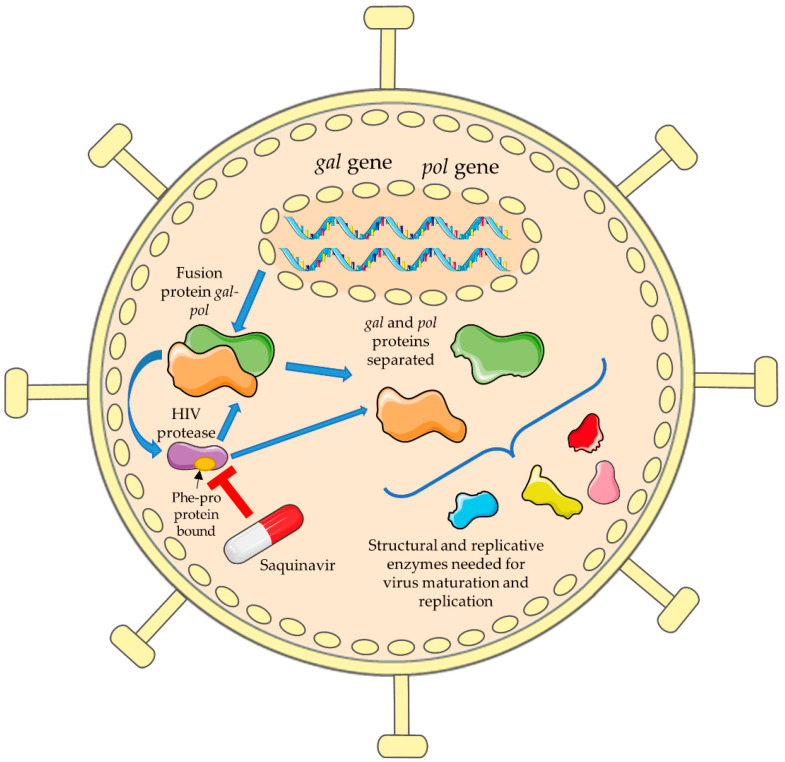
Mechanism of action of saquinavir in the HIV. Viral replication in the HIV can only happen after the translation of a fusion protein between the *pol* and *gag* genes. The HIV protease is formed here, where it cleaves itself from this big fusion protein and then cleaves the fusion protein into precursor proteins and then into structural and replicative enzymes. Saquinavir is a mimetic of the Phe–Pro peptide bond, which binds strongly to the protease, leading to competitive inhibition of its activity (this figure was partly generated using Servier Medical Art provided by Servier licensed under a Creative Commons Attribution 3.0 unported license).

In terms of the mode of action, as previously mentioned, saquinavir is a protease inhibitor. The HIV protease is very important because some of the replicative enzymes needed for viral replication in the HIV can only be formed by translation of a fusion protein between the *pol* and *gag* genes. This HIV aspartic protease is formed here, cleaving itself from this big *gag–pol* fusion protein. Afterwards, it will cleave the precursor proteins present in the *gag–pol* fusion protein into structural and replicative enzymes [14]. A virus with the HIV protease inhibited or mutated cannot process the *gag–pol* fusion protein, leading to the formation of an immature provirus without the capacity to infect cells, taking away the infectious nature of the HIV [15]. In terms of cleavage sites, the HIV protease cleaves the *gag–pol* fusion protein at tyrosine–proline (Tyr–Pro) or phenylalanine–proline (Phe–Pro) residues, and the process involves a high-energy tetrahedral transition state consisting of the addition of water to an amide bond. Saquinavir was designed as a mimetic transition state of the Phe–Pro peptide bond, which binds strongly to the protease enzyme, leading to competitive inhibition of its activity needed for virus maturation and proliferation [16]. This mechanism of action is demonstrated in Figure 2.

## 3. Saquinavir for COVID-19 Treatment

Coronavirus disease 2019 (COVID-19) emerged in late 2019 and resulted in a worldwide pandemic that led to and still is causing millions of confirmed cases and deaths. This disease is associated with severe acute respiratory syndrome coronavirus 2 (SARS-CoV-2) which spreads rapidly through respiratory droplets or contaminated surfaces [17]. The SARS-CoV-2 virus contains two open reading frames in its DNA that encode two polyproteins 1a and 1b, which are then processed to produce 16 nonstructural proteins needed for the virus to complete its lifecycle. In betacoronaviruses, like SARS-CoV 2, the enzymes responsible for this process are the chymotrypsin-like protease (3CLpro) and, to a lesser extent, the papain-like protease (PLpro), which is why these are the main targets for COVID-19 treatment [18].

The COVID-19 disease is characterized by several symptoms, from mild ones, like fever, fatigue, and cough, to more severe lung-related symptoms, such as acute respiratory distress syndrome (ARDS) and pneumonia, that lead to hospitalizations and deaths [19]. Since COVID-19 was quickly spreading there was a pressing need for the development of a therapy for it. The development of drugs, however, could take years and it has a high cost and a low success rate, which is why one of the first strategies implemented was the repurposing of the already existing drugs, namely antivirals, to try to find ones that could be used for COVID-19 [20]. Here, we discuss the works related to the repurposing of saquinavir for this disease.

An in silico study tried to discover a potential drug for COVID-19 treatment. The authors used an approved compound database that included the major bioactive pharmaceutical compounds, which had over 3000 drugs. A docking model of the 3CL protease was then used as well. This protease is highly conserved from SARS to SARS-CoV-2, the former of which has histidine residue His162 that when mutated leaves the enzyme inactive. Due to high homology, the 3CL protease also has a similar residue, His163, as well as a second histidine residue that also interacts with ligation bounds. These residues were used in molecular docking calculations to understand if any of the drugs had the potential to inhibit this protease. Saquinavir was among the several hits obtained for antiviral drugs [21]. Indeed, several studies throughout the two years since the pandemic started have shown through in silico methods like molecular docking, several linear and nonlinear models, virtual screening technology, and others that saquinavir is a promising drug for repurposing and use against SARS-CoV-2 due to its affinity with the 3CL protease, which causes its inhibition and halts SARC-CoV-2 maturation [22,23,24,25]. 

**Figure 3:**
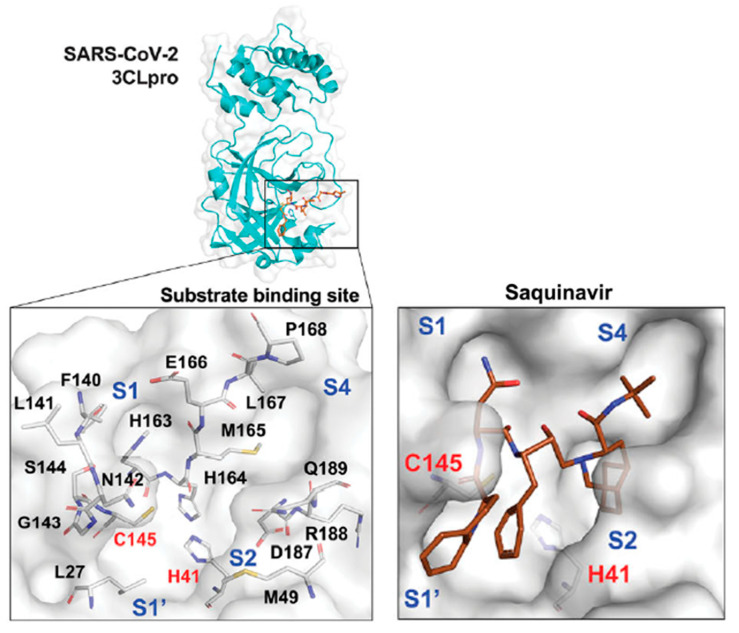
Proposed model of inhibition of the SARS-CoV-2 3CL protease by saquinavir (PDB ID 6LU7). This protease has four subsites in the substrate-binding site (S1–S4), with the catalytic residues C145 and H41 (Cys–His catalytic dyad) needed for 3CLpro activity (left). Saquinavir binds with all four subsites and with the catalytic residues, causing an inhibitory effect (right). Adapted from [26].

A similar study was conducted in vitro with around 774 FDA-approved drugs. Inhibition of 3CLpro after incubation of 0–100 μM was assessed using a fluorogenic peptide as a substrate, and a dose response graph and an IC_50_ were obtained. Among the seven drugs with the highest inhibitory activity was saquinavir, with an IC_50_ of 9.92 ± 0.73 μM, even despite it being an aspartic protease inhibitor and 3CLpro being a cysteine protease. To understand how these drugs inhibited 3CLpro, molecular docking was performed to simulate the binding model of this enzyme. Saquinavir filled all four subsites of 3CLpro in the substrate-binding site, binding to the catalytic residues C145 and H41, the so-called Cys–His catalytic dyad that is responsible for the activity of this protease, causing the inhibitory effect. All this combined demonstrated the potential of saquinavir for COVID-19 treatment [26]. The proposed mechanism of binding of saquinavir on this SARS-CoV-2 protease can be seen in Figure 3. 

**Figure 4:**
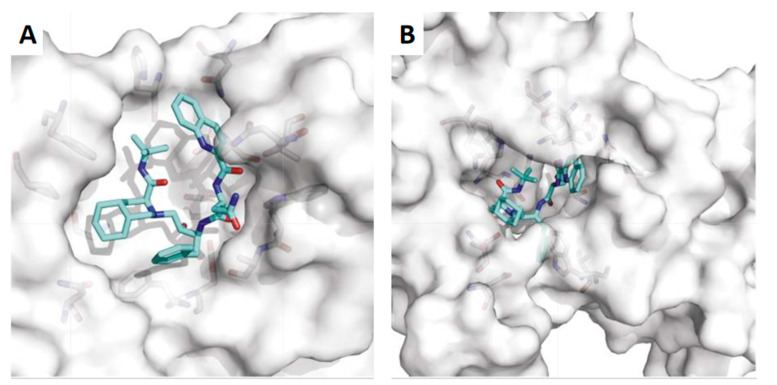
Proposed model of inhibition of the SARS-CoV-2 NSP14 by saquinavir (PDB ID 5NFY). (**A**) Saquinavir interaction with the *N*-terminus of NSP14; (**B**) saquinavir interaction with the C-terminus of NSP14. Adapted from [28].

Another target for the treatment of COVID-19 could be one of the 16 nonstructural proteins (NSP). NSP14, for example, has a 3′-5′ exoribonuclease domain that corrects mismatched nucleotides during SARS-CoV-2 replication. It also has aт S-adenosyl methionine (SAM)-dependent (guanine-N7) methyl transferase (N7-MTase) that is involved in the synthesis of a cap structure on the 5′ end of SARS-CoV-2 mRNA, which helps to evade the host’s defenses and during the translation process [27]. With this in mind, a study tried to determine which drugs could target NSP14 and possibly inhibit the SARS-CoV-2 virus. Firstly, the virus and the SARS NSP14 amino acid sequences were obtained and aligned so as to use the SARS NSP14 as a template to model the SARS-CoV-2 NSP14, of which several structures were constructed. Then, a docking box (target) was decided for the best structure, and the binding energy of each drug with the docking box of NSP14 was calculated. Saquinavir was suggested to bind with the C- and *N*-terminus active pockets of NSP14 and interact with the key amino acid residues in its active center, with a strong binding free energy. Taken together, saquinavir can have an inhibitory effect in NSP14, which would leave SARS-CoV-2 more vulnerable to lethal mutagenesis and host’s defenses [28]. The proposed mechanism of binding of saquinavir to the SARS-CoV-2 NSP14 can be seen in Figure 4.

**Figure 5:**
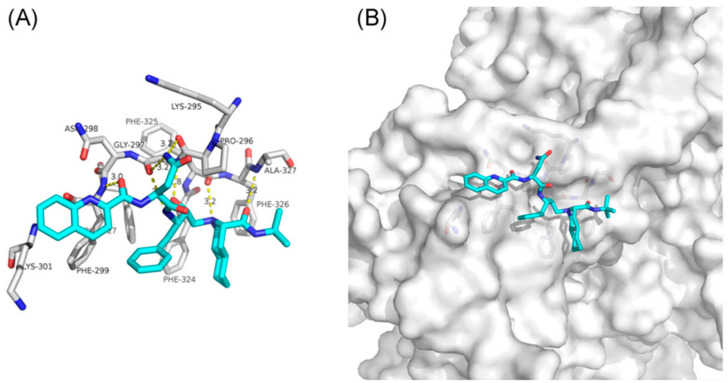
Proposed model of inhibition of SARS-CoV NSP12–NSP7 by saquinavir (PDB ID 6NUR). (**A**) Saquinavir interaction with the interface of the crystal structure of NSP12–NSP7; (**B**) saquinavir binding in the protein interface pocket between NSP12 and NSP7. Adapted from [29].

Another target in this vein could be the NSP12–NSP7–NSP8 complex, in which NSP12 is essential for RNA-dependent RNA polymerase synthesis (RdRp) needed for replication. For this, NSP12 has to bind with NSP7 and NSP8, and disrupting this could mean a way to inhibit RdRp activity and consequent replication of SARS-CoV-2. To test this theory, the SARS-CoV and SARS-CoV-2 NSP12 crystal structures were targeted for molecular docking of several drugs, with the docking box being in the NSP7 and NSP8 binding pockets, the active sites of NSP12 that allows for its activity. Saquinavir was also found to be able to bind with the SARS-CoV NSP7 and NSP12 interface and act as an interfacial blocker. Since the SARS-CoV-2 NSP7 and NSP12 proteins have a 98.8% and 96.35% similarity with those of SARS-CoV, respectively, the effect of saquinavir could also be translated to SARS-CoV-2, showing that this drug can have similar effects on the inhibition of replication of the COVID-19-causing virus [29]. The proposed mechanism of binding of saquinavir to the SARS-CoV NSP12–NSP7 complex can be seen in Figure 5.

Despite these findings showing that saquinavir is a potential inhibitor of several SARS-CoV-2 components, like the main protease (3CLpro), RdRps, and nonstructural proteins, and could be a potential drug for COVID-19 treatment, as far as we are aware, no clinical trial to assess this in humans has been started as of the writing of this article.

## 4. Repurposing of Saquinavir for Cancer Treatment

Recently, a new strategy for the discovery of new therapies has emerged called drug repurposing, which involves using already approved drugs for a new indication. This comes with several advantages, such as the fact that the drug is already proven to be safe for human use, it quickens the drug development time, and it also requires fewer funds [30]. With this in mind, several researchers have tried to understand if saquinavir could be repurposed from its use as an antiviral to use for cancer treatment. 

The first indication that saquinavir could have anticancer effects was when it was found that it caused regression of Kaposi sarcoma (KS). This is a vascular tumor that is associated with infection by Kaposi sarcoma herpesvirus/human herpesvirus 8, occurring mainly in mucocutaneous sites [31]. It is characterized by angiogenesis, the appearance of spindle cells, the tumor cells of KS, and inflammatory cell infiltration [32]. To test this hypothesis, a study was conducted using in vitro and in vivo models of KS tumor growth and lesion formation (without the virus), angiogenesis, and nude mice infected with KS cells, all incubated with saquinavir. The results showed strong inhibition of angiogenic lesions in vivo, with reduction of angiogenesis and spindle cell growth by saquinavir, to a degree comparable to paclitaxel, a reference anticancer drug used for KS therapy. This was caused by inhibition of cell invasion essential for angiogenesis, which was accompanied with an antitumor growth effect. Taken together, the study demonstrated an antiangiogenic, antitumor, and anti-KS effect of saquinavir, an early indication of its potential for repurposing for cancer treatment [33]. 

Another example of what has been studied is the use of saquinavir in cervical cancer. The main agent that causes cervical cancer is the persistent infection by high-risk human papillomaviruses (HR-HPVs), which produce oncoproteins (mainly E6 and E7) that alter the cell cycle regulators of cervical cells [34]. HIV-PIs have been thought to potentially treat HR-HPV infection by regulating E6 and E7 oncoprotein turnover, which could assist in the treatment of cervical cancer. This was tested using various primary and immortalized cervical cancer cell lines (CC1, CC2, HeLa, CaSki, HT3, and C33a) incubated with several HIV-PIs, namely saquinavir, in concentrations ranging from 5 to 80 μM. Saquinavir had the highest inhibiting effect on cell proliferation in all cell lines at all concentrations, with an IC_50_ of 19 μM at 96 h in HeLa cells. It also proved to be highly inhibitive of the activity of various proteasomes, albeit needing a higher concentration than the one needed for cell proliferation inhibition (60–80 μM). This was not translated to a strong alteration in cell cycle phases, only slowing the process. Using the IC_50_, saquinavir interfered with clonogenicity and cell invasion, demonstrating a proteasome-independent inhibitory effect and suggesting other oncogenic pathways. This shows the potential of saquinavir for the treatment of cervical cancer patients, even if not through proteasome interference, but still requires a study in vivo to understand if these effects only happen in vitro or not [35].

Cancer cells have mechanisms that allow them to escape apoptosis. One example is the limitation of tumor necrosis factor alpha (TNF-α), which is an inducer of apoptosis, by coactivation of nuclear factor kappa B (NF-κB) [36]. This only happens if its inhibitor, IκBα, is phosphorylated, ubiquitinated, and degraded by the multicatalytic proteasome 26S, which allows for nuclear translocation of NF-κB and consequent gene expression. Inhibition of proteasome 26S has been shown to induce apoptosis and radiosensitization in cancer cells [37]. Since the 20S core unit of the mammalian proteasome and the HIV-1 protease share cleavage locations, HIV-PIs could be used to inhibit the 26S proteasome and induce apoptosis in cancer cells, something that was tested firstly with ritonavir and then with saquinavir [38,39]. To test the latter, multiple cancer cell models were used, namely glioblastoma cells (U373), erythroleukemia (K562) and Jurkat leukemia cells, human prostate carcinoma cell lines (PC-3, LnCaP, and DU-145), murine macrophages (RAW 264.7), and human bladder carcinoma cells (ECV 304), exposed to increasing concentrations of saquinavir (0–100 μM). Saquinavir blocked NF-κB activation and stabilized IκBα on PC-3 and macrophage cells in a concentration-dependent way, and also inhibited the 20S and 26S proteasome function similarly in PC-3 and ECV 304 cells, but with a high IC_50_ (50 μM), probably due to saquinavir being a substrate of P-gp. As was expected, the tested cells (PC-3, DU-145, K562, U373, and Jurkat leukemia lines) suffered apoptosis at concentrations similar to the ones that caused proteasome inhibition, confirming the hypothesis that inhibition of proteasome and NF-κB activation by saquinavir leads to apoptosis in cancer cells. Other than this, saquinavir also radiosensitized prostate cancer cells to ionizing radiation, a consequence of proteasome function inhibition. This shows that saquinavir can cause apoptosis and radiosensitization of several types of cancer cells by proteasome inhibition [38].

A new approach for cancer treatment while using repurposed drugs is combining them with anticancer drugs, allowing for the targeting of multiple pathways using lower doses and, consequently, decreasing toxicity while having an additive or, ideally, a synergistic effect [40]. Chronic myelogenous leukemia (CML) is a type of cancer associated with hematopoietic stem cells and occurs due to the creation of the chimeric gene BCR-ABL that produces a tyrosine kinase responsible for excessive proliferation [41]. Treatment for this neoplasia was greatly improved with the synthesis of imatinib, a potent inhibitor of the tyrosine kinase previously mentioned. However, this drug does not eradicate cells expressing this BCR-ABL gene, which leads to resistance of CML to treatment [42]. BCR-ABL also leads to activation of NF-κB, which, as mentioned before, protects cancer cells, in this case, tumorous hematopoietic cells, from apoptosis and cell death, being an important component in BCR-ABL tumorigenicity [36,43]. Since saquinavir has been shown to inhibit NF-κB [38], the study of it combined with imatinib in sensitive and resistant CML cell lines was performed to understand if it could have a positive combination effect. After 72 h, saquinavir alone inhibited proliferation and caused apoptosis in a time- and concentration-dependent fashion, at lower concentrations (6–9.6 μM) in resistant cell lines, the concentrations corresponding to the ones used and well-tolerated in HIV treatment. More importantly, the combination of imatinib with 5 μM of saquinavir led to a higher activity of imatinib in all cell lines, with a decrease in the IC_50_ of imatinib when in combination (from 1–19 μM to 0.03–0.81 μM). This clearly displays the potential of the combination of imatinib, an antineoplastic drug, with repurposed saquinavir in the treatment of CML [44]. This combination has been further explored successfully in another type of cancer, neuroblastoma [45]. This is an embryonal malignancy of the sympathetic nervous system that affects the development of paravertebral sympathetic ganglia and adrenal medulla in the early stages of childhood, and it is one of the major solid tumors in pediatric patients [46]. It has been shown that imatinib can inhibit the growth of neuroblastoma cells (in vitro) and tumors (in vivo) since imatinib is an inhibitor of certain cell receptors expressed by these cells that could be targeted for neuroblastoma treatment [47]. However, when this was translated to a phase II clinical trial with refractory or relapsed neuroblastoma tumors that express the receptors mentioned, no significant results were obtained, contrary to what was expected [48]. Since saquinavir in combination with imatinib enhances its activity for CML, the same was tested with several neuroblastoma cell lines. Saquinavir alone had an antiproliferative effect on these cells, with a lower IC_50_ (3.8 μM after 72 h) than that obtained for CML, and 5 μM of saquinavir enhanced imatinib activity, a result obtained with both drugs at clinically achievable concentrations. The combination with saquinavir also increased the anti-invasive effect of imatinib on neuroblastoma cells. Saquinavir had proapoptotic activity (20 μM) which was enhanced by the combination with imatinib and in the presence of serum (10–15 μM). The mechanism of action behind the saquinavir effect is associated, at least partially, with the NF-κB and 26S proteasome inhibition observed in this study when using low concentrations. The additive activity of saquinavir and imatinib suggests that this combination could be useful in neuroblastoma treatment, with a strong focus on remission maintenance [45].

Maintaining protein homeostasis is a process required for cell survival, with a key factor being making sure that proteins are correctly folded and assembled and that protein synthesis proceeds without errors. For the former, the heat shock response pathway helps in protein folding, and for the latter, the proteasome degrades any incorrectly translated protein, a process even more important in cancer cells due to the high protein synthesis that is associated with them [49]. Accumulation of un/misfolded or defective proteins leads to proteotoxic stress. When this happens in the endoplasmic reticulum (ER), it causes ER stress, a type of proteotoxic stress, and activation of the unfolded protein response (UPR), increasing the synthesis of chaperone proteins while shutting down protein synthesis and accelerating protein degradation [50]. When unfolded proteins accumulate beyond the capacity of the cells, UPR triggers the expression of proapoptotic factors that lead to cell death [51]. The induction of proteotoxic stress and consequent apoptosis could be a target for cancer treatment and could be achieved by a combining a proteasome inhibitor so that defective proteins accumulate and a UPR inductor to provoke apoptosis [52]. This theory was tested in acute myeloid leukemia cells to see if HIV-PIs, like saquinavir, could sensitize cells to proteasome inhibitors, like bortezomib. Saquinavir proved to have a cytotoxic effect at low concentrations in all stages of differentiation or lineage. The combination proved to be synergic, an effect provoked by proteotoxic stress-induced UPR activation. It can be concluded then that saquinavir (and other HIV-PIs) could be used in proteotoxic stress-targeted therapy for acute myeloid leukemia [53]. 

Another mechanism that allows cancer cells to withstand treatments is resistance to ionizing radiation. One of the pathways known to assist in this is the phosphatidylinositol 3-kinases (PI3K)—protein kinase B (Akt) pathway, usually overexpressed in cancer cells compared with normal cells [54]. Ionizing radiation-activated PI3K phosphorylates lipids, producing secondary messengers that bind with Akt, which is then translocated to the plasma membrane, where it is activated and controls cancer cell survival and several other processes, like metabolism and cell cycle, ultimately increasing resistance to radiation the more Akt is activated [55]. Due to this, inhibition of the PI3K–Akt pathway is a possible target to radiosensitize cancer cells, which could be achieved using HIV-PIs. A study was then performed using several types of cancer cell lines, namely bladder (T24), head and neck (SQ20B), pancreatic (MIAPACA2), lung (A459), and rat fibroblasts as well as nude mice to test five types of PIs, saquinavir being one of those. Saquinavir decreased Akt phosphorylation at 25 μmol/L in a time-dependent manner in head and neck SQ20B cells, with a total loss of detection after 20 min, having similar effects at lower concentrations (5–10 μmol/L) only after 24 h. Cell death happened after 2 h with 25 μmol/L, after 24 h with 10 μmol/L, and after 48 h with 1 μmol/L. Despite the high-toxicity-affecting assays, radiosensitization was obtained in T24 bladder cancer cells after Akt inhibition. They concluded then that saquinavir, along with nelfinavir and amprenavir, inhibited Akt phosphorylation and serum concentrations, which then left cancer cells sensitized to radiation [56]. This inhibition effect was further tested in vivo with patients taking these antiretrovirals as HIV treatment and patients with different regimens using a biomarker for Akt activation (phospho-Akt) in peripheral leukocytes. Patients taking saquinavir (and nelfinavir) had lower levels of phosphor-Akt in leukocytes than patients with other therapies, which could be translated to other tissues and tumors and would suggest radiosensitization. This was achieved without an increase in secondary toxic effects related to radiation for the patients. As such, saquinavir could be used safely as a radiosensitizing agent for cancer cells in combination with radiation [57]. Taking these results and the ones from the 26S proteasome study mentioned above [38], it is easy to see that saquinavir has a radiosensitizing effect on tumor cells and could be used as an anticancer drug for that purpose.

From all the works mentioned above, several mechanisms by which saquinavir can have anticancer effects have been discovered, from 20S and 26S proteasome inhibition to radiosensitization and inactivation of NF-κB (and consequent induction of apoptosis). An overview of all the works can be seen in Table 1. Despite all these works, no further clinical investigation has taken place as of the writing of this article. However, there are other works that indicate that saquinavir could be important and worth exploring in other diseases, such as in the UPR pathway in Alzheimer’s disease and diabetes [58,59], in rheumatoid arthritis [60], or even in malaria [61] and tuberculosis [62].

## 5. Conclusions

Saquinavir has been of great importance in HIV treatment, allowing for the longevity of patients’ life since its approval. Throughout the years, there have been several works on the use of this drug in cancer treatment, but no further research in clinical trials with humans. Recently, the emergence of COVID-19 and the need for the development of therapy has shined a new light on the repurposing of saquinavir, mainly in the inhibition of the SARS-CoV-2 protease, with success both in silico and in vitro. Despite no further clinical investigation taking place, the results in protease inhibition are also translated into a proteasome inhibition in cancer cells. The combination of these similar pharmacological pathways and also the development and improvement of more accurate in silico models used for COVID-19 repurposing can also be used in cancer repurposing of saquinavir, which can lead to its approval for this use in the future. 

## Figures and Tables

**Table 1:** Works on the repurposing of saquinavir for anticancer treatment.

Cancer type	Model	Main results	Ref.
Kaposi sarcoma	In vitro models of KS tumor growth and lesion formation and nude mice	Inhibition of angiogenic lesions in vivo (reduction of angiogenesis and spindle cell growth), cell invasion, and antitumor growth effect	[33]
Cervical cancer	CC1, CC2, HeLa, CaSki, HT3, and C33a cell lines	High inhibitory effect in all cell lines (IC_50_ of 19 μM at 96 h in HeLa cells) independent of the proteasome. Interference with clonogenicity and cell invasion	[35]
Prostate cancer	PC-3, LnCap, and DU-145 prostate cancer cell lines	Blockage of NF-κB activation and stabilization of IκBα due to inhibition of the 20S and 26S proteasomes (at higher concentrations). Consequent induction of apoptosis (at the concentrations similar to the ones that cause proteasome inhibition). Radiosensitization to ionizing radiation	[38]
Bladder cancer	ECV 304 human bladder carcinoma cell line	Inhibition of the 20S and 26S proteasomes with a higher IC_50_ (50 μM) (since saquinavir is a substrate of P-gp)	
Leukemia	Jurkat leukemia cells	Induced apoptosis when using concentrations that inhibit the 20S and 26S proteasomes	
Erythroleukemia	K562 erythroleukemia cell line		
Glioblastoma	U373 glioblastoma cell line		
Chronic myelogenous leukemia	Sensitive and resistant CML cell lines	Time- and concentration-dependent inhibition of proliferation and induction of apoptosis (alone). Improvement of imatinib activity in all cell lines when in combination with saquinavir (decreasing its IC_50_)	[44]
Neuroblastoma	Neuroblastoma cell lines	Antiproliferative effect with low concentrations (alone) and enhancement of imatinib’s activity in combination (both antiproliferative and anti-invasive effect). Enhanced apoptotic effect of saquinavir when in combination. NF-κB and 26S proteasome inhibition	[45]
Acute myeloid leukemia	Acute myeloid leukemia cell lines	The cytotoxic effect at low concentrations and in all stages of differentiation. Synergic combination with bortezomib (PI), causing proteotoxic stress due to UPR activation	[53]
Head and neck cancer	SQ20B head and neck cell line	Decreased Akt phosphorylation in a time- and concentration-dependent way, with total loss at 20 min at lower concentrations at 24 h and with higher concentrations. Cell death effect	[57]
Bladder cancer cells	T24 bladder cancer cell line	Akt inhibition and consequent radiosensitization of cells	

## Data Availability

Not applicable.
